# Electrical Phenomena in the Alps

**Published:** 1863-11

**Authors:** 


Electrical Phenomena in the Alps.—A party consisting of three Englishmen and a lady, with two guides, endeavored to make the ascent of the Alps on the 10th of July last, but when they had proceeded some distance they were prevented by a storm, during which they got into the middle of an electric cloud. Their hair emitted a hissing, crackling sound, as if it had been under the influence of a powerful electrical machine, and over their faces and bodies they experienced a pricking, burning sensation. There were peals of thunder heard, at each of which the party received an electric shock, but no lightning was seen. The right arm of one of the party was paralyzed for several minutes; the snow emitted hissing sounds; the veil worn by the lady stood straight out, as also did the hair of all of them; which looked so ludicrous, that they could not help bursting into laughter. These phenomena lasted for about twenty-five minutes, and no evil effects were felt afterwards.—*Scientific American.*

